# Construction of a Global Pain Systems Network Highlights Phospholipid Signaling as a Regulator of Heat Nociception

**DOI:** 10.1371/journal.pgen.1003071

**Published:** 2012-12-06

**Authors:** G. Gregory Neely, Shuan Rao, Michael Costigan, Norbert Mair, Ildiko Racz, Giedre Milinkeviciute, Arabella Meixner, Swetha Nayanala, Robert S. Griffin, Inna Belfer, Feng Dai, Shad Smith, Luda Diatchenko, Stefano Marengo, Bernhard J. Haubner, Maria Novatchkova, Dustin Gibson, William Maixner, J. Andrew Pospisilik, Emilio Hirsch, Ian Q. Whishaw, Andreas Zimmer, Vaijayanti Gupta, Junko Sasaki, Yasunori Kanaho, Takehiko Sasaki, Michaela Kress, Clifford J. Woolf, Josef M. Penninger

**Affiliations:** 1 Neuroscience Program, Garvan Institute of Medical Research, Sydney, New South Wales, Australia; 2 School of Biotechnology and Biomolecular Sciences, University of New South Wales, Sydney, New South Wales, Australia; 3 IMBA, Institute of Molecular Biotechnology of the Austrian Academy of Sciences, Vienna, Austria; 4 F. M. Kirby Program in Neurobiology, Children's Hospital Boston and Department of Neurobiology, Harvard Medical School, Boston, Massachusetts, United States of America; 5 Division of Physiology, Department of Physiology and Medical Physics, Innsbruck Medical University, Innsbruck, Austria; 6 Laboratory of Molecular Neurobiology, Department of Psychiatry, University of Bonn, Bonn, Germany; 7 Strand Life Sciences, Bangalore, India; 8 Department of Anesthesia and Critical Care, Massachusetts General Hospital and Harvard Medical School, Boston, Massachusetts, United States of America; 9 Molecular Epidemiology of Pain Program, Department of Anaesthesiology, University of Pittsburgh, Pittsburgh, Pennsylvania, United States of America; 10 Yale Center for Analytical Sciences, Yale University, New Haven, Connecticut, United States of America; 11 Center for Neurosensory Disorders, University of North Carolina at Chapel Hill, Chapel Hill, North Carolina, United States of America; 12 Molecular Biotechnology Center, Department of Genetics, Biology, and Biochemistry, University of Torino, Torino, Italy; 13 Max Planck Institute of Immunobiology, Freiburg, Germany; 14 NeuroDetective, Lethbridge, Alberta, Canada; 15 Department of Medical Biology, Akita University Graduate School of Medicine, Akita, Japan; 16 Department of Physiological Chemistry, Graduate School of Comprehensive Human Sciences and Institute of Basic Medical Sciences, University of Tsukuba, Tsukuba, Japan; 17 Research Center for Biosignal, Akita University, Akita, Japan; The Wellcome Trust Centre for Human Genetics, University of Oxford, United Kingdom

## Abstract

The ability to perceive noxious stimuli is critical for an animal's survival in the face of environmental danger, and thus pain perception is likely to be under stringent evolutionary pressure. Using a neuronal-specific RNAi knock-down strategy in adult *Drosophila*, we recently completed a genome-wide functional annotation of heat nociception that allowed us to identify α2δ3 as a novel pain gene. Here we report construction of an evolutionary-conserved, system-level, global molecular pain network map. Our systems map is markedly enriched for multiple genes associated with human pain and predicts a plethora of novel candidate pain pathways. One central node of this pain network is phospholipid signaling, which has been implicated before in pain processing. To further investigate the role of phospholipid signaling in mammalian heat pain perception, we analysed the phenotype of PIP5Kα and PI3Kγ mutant mice. Intriguingly, both of these mice exhibit pronounced hypersensitivity to noxious heat and capsaicin-induced pain, which directly mapped through PI3Kγ kinase-dead knock-in mice to PI3Kγ lipid kinase activity. Using single primary sensory neuron recording, PI3Kγ function was mechanistically linked to a negative regulation of TRPV1 channel transduction. Our data provide a systems map for heat nociception and reinforces the extraordinary conservation of molecular mechanisms of nociception across different species.

## Author Summary

Nociception is the perception of noxious, potentially damaging stimuli; and this pain or its equivalent behavioral readout is evolutionarily conserved from fruit flies to humans. Using genetic techniques in the fruit fly, we have been able to evaluate the potential functional contribution of every gene in the fruit fly genome for a role in avoidance of high noxious temperatures (heat pain-like responses). Using this functional genomics data set, we have developed a conserved network map of heat pain/nociception that predicts numerous conserved genes and pathways as novel pain pathways, including phospholipid signaling. Studies in multiple mutant mice confirmed a role for lipid signaling in pain perception, and more specifically we identify the critical lipid kinase (PI3Kγ) as a negative regulator of TRPV1 (receptor for noxious heat and capsaicin, the active component in chili peppers) signaling. This finding shows that our fly-based genetic pain network map is a valuable tool for the discovery of novel “nociception genes” in mammals.

## Introduction

Although studies in inbred mouse strains and in human twin cohorts have indicated that pain has a strong genetic component [1]–[5], with an estimated heritability of ∼50%, little is known about the specific genes involved in regulating pain sensitivity across phyla. Further, conservation of gene function between species and across evolutionary time acts as a useful tool to develop an understanding of core genetic mechanisms relative to more specialized programs and how they influence behavior [6]. *Drosophila* is an excellent model organism for characterizing genetic regulators of behavior such as nociception [7]. Use of *Drosophila* genetics has highlighted a conserved role for multiple genes in the detection and avoidance of noxious heat [8], [9], and recent work on mechanosensation suggests the genetics of this process is likely also highly conserved across phyla [10]. We have previously reported a global *in vivo* RNAi screen for avoidance of noxious heat in *Drosophila*, and identification of hundreds of novel genes required in the adult fly, for its manifestation [8]. To interrogate this resource, we have now constructed a global systems network of heat pain. Our goal was to identify potentially conserved genes and pathways involved in pain perception, to provide a tool for focusing research on key pain molecular pathways.

One pathway highlighted in this global systems network was phosphatidylinositol signaling. Phosphatidylinositol signaling is a second messenger cascade involving the sequential phosphorylation of phosphatidylinositol 4-phosphate (PIP) to generate phosphatidylinositol 4,5-bisphosphate (PIP2) via PIP5' kinases (phosphatidylinositol-5-OH kinase; PIP5K), and then phosphorylation of PIP2 via PI3 kinases (phosphatidylinositol-3-OH kinase; PI3K) to generate phosphatidylinositol 3,4,5-trisphosphate (PIP3). In mammalian systems phospholipid signaling has been implicated in regulating pain perception [11]–[13], TRPV1 function [14]–[21], and itch [22], but how phosphatidylinositol signaling is involved in mammalian nociception is controversial, with data suggesting for example that PIP2 may either increase or decrease TRPV1 function [23], [24]. Based on the suggested involvement of phospholipid signaling in our conserved functional pain network, and in context of the controversial role for phosphatidylinositol signaling in pain perception, we employed genetic approaches to evaluate the role of phosphatidylinositol signaling in mammalian nociception.

## Results/Discussion

To construct a global systems network of heat pain we first identified potential mouse and human orthologs of fly candidate pain genes (Figure S1). Of the 580 candidate fly thermal nociception genes we had previously identified [8], 399 had human orthologs (Table S1), many of which are known mammalian pain genes (Table S2). Gene ontology (GO) analyses of the human and mouse orthologs of the fly thermal nociception hits showed a marked enrichment of genes involved in neurotransmission and secretion, housekeeping systems such as mitochondrial structure, ATP synthesis, metabolism, or calcium signaling (Figure S2A–S2C and Table S3). We next generated an interaction map based on first-degree binding partners for the fly thermal nociception hits (Table S4). All binding partners were identified in yeast-2-hybrid screens and reported in the biomolecular interaction network database BIND, i.e. binding partners experimentally confirmed to interact with the candidate genes. Among the first degree binding partners in this interaction network, we found fly allatostatin C receptor 1, which has homology with mammalian opioid receptors, a fly homolog of Lmx1b, which regulates central serotonergic responses to opioids [25], a fly gene related to the nuclear factor-erythroid 2-related factor 2, which has anti-nociceptive effects [26] by inducing upregulation of heme oxygenase-1, and tyrosine hydroxylase, the enzyme required for dopamine and catecholamine production [27]. Thus, our genome-wide functional screen for thermal nociception in flies has generated a human gene network that includes orthologs of several known mammalian pain genes, in addition to numerous uncharacterized genes and pathway not previously associated with pain perception.

**Figure 1:**
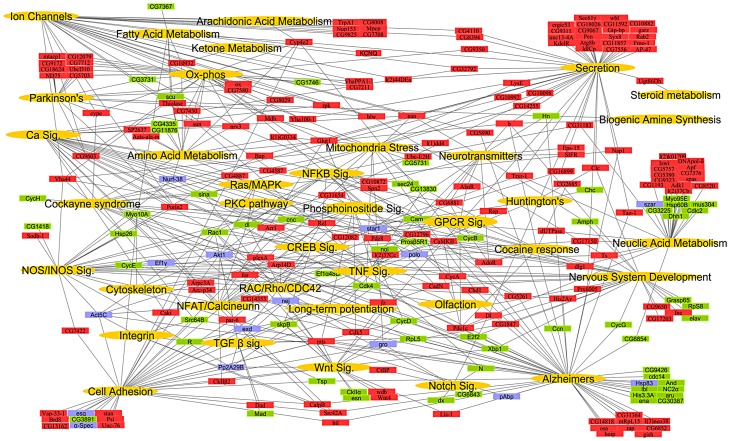
A global network map of thermal nociception. The systems network includes data from significantly enriched *Drosophila* KEGG pathways and GO processes, mouse and human KEGG pathways and C2 gene sets. Pathways, processes and gene sets that share a role in a biological process were pooled into functional classes while the underlying genes that constitute them are depicted with a connection to their respective functional class. Functional classes (gold), genes representing direct hits with a thermal nociception phenotype (red), their first degree binding partners (green), and developmental lethal genes (blue) represent the nodes in the network. Only select KEGG pathways, biological processes and C2 gene sets were used to build systems map. For the entire list of individual pathways, gene sets, and processes see Tables S5, S6, S7.

To construct a mammalian systems map of thermal nociception, we performed an enrichment analysis (using KEGG pathways and Broad Institute C2 gene sets) on the mouse and human orthologs of the fly pain genes and their first degree binding partners (Figure S1; Table S5 and Table S6). We found significant enrichment of genes (hypergeometric enrichment >90%) involved in mitochondria, metabolism, calcium signaling, inflammation, cell adhesion, RNA processing, and neurotransmission. Finally, to generate a comprehensive conserved network map of thermal nociception, the KEGG pathways from *Drosophila*, mouse and human were combined with relevant gene sets from the C2 annotations to create a global putative “nociception network” (Figure 1; Table S7). From this combined systems network, we identified gene sets or pathways known to play key roles in many major neural systems.

The connectivity of the entire systems map remains intact even after omitting all binding partners (Figure S3), i.e., expansion of the “pain” network map by including binding partners does not introduce a bias. Moreover, all except one pathway in the network map has >50% representation from the *Drosophila* heat pain hits alone. To test whether this approach can predict mammalian pain genes, we measured the overlap of the direct candidate pain hits and their binding partners with previous rat microarray expression profiling data generated from pain studies [28] and all “pain” annotated genes from OMIM (Online Mendelian Inheritance in Man, NCBI). Intriguingly, we found a 38.55% overlap of our direct pain hits and a 42.33% overlap of their binding partners with the microarray and OMIM data for pain. In contrast, 100 random gene lists gave a maximum of 6% overlapping genes and a minimum of 0% (Table 1; Table S8). Thus, our hypothesis-free systems map is markedly enriched for genes known to be associated with rodent and human pain. Considering the complexity of the neuronal network involved in generating nociception in the periphery and CNS, these pathways may operate across several neurons; however, our genome-wide functional fly pain screen and *in silico* data mining provide a road map for conserved molecular components and pathways putatively involved in heat nociception globally across phyla.

**Table 1:** Comparison of systems map with microarray and OMIM data for pain.

	Genes in system map		Genes matching with pain report in OMIM		Genes matching with pain report in Microarray data		Common to OMIM & Microarray	Total matched genes (Microarray+OMIM)	
	Number of genes in System map	Percentage of genes in System map	Number of genes in OMIM	Percentage of genes in OMIM	Number of genes in Microarray data	Percentage of genes in Microarray data	Number of genes common to OMIM & Microarray	Number of genes in Microarray+OMIM	Percentage of genes in Microarray+OMIM
**Pain**	166	68.03%	40	24.10%	34	20.48%	10	64	38.55%
**Binding Partner**	78	31.97%	20	25.64%	23	29.48%	10	33	42.31%

A comparison of genes in the global network map of thermal nociception with genes with a pain annotation in the OMIM database (http://www.ncbi.nlm.nih.gov/omim) and previous rat microarray expression profiling data generated from pain studies [28] showed the global network map of thermal nociception is enriched for genes implicated in mammalian pain diseases.

We next wanted to validate whether this “nociception network” has the power to identify conserved pathways involved in nociception, and if this pathway information can then help to pinpoint key mammalian pain genes. To this end we focused our first efforts on phosphatidylinositol signaling, one of the major nodes predicted from the pain systems map, and a heat pain precedented pathway. Phosphatidylinositol signaling has been implicated in heat nociception and regulation of TRPV1 by multiple groups [11], [13], [16], [18], however its precise role has been controversial [16], [18], [23], and the specific participation of different phospholipid kinases has never been evaluated genetically, which we now decided to do.

**Figure 2:**
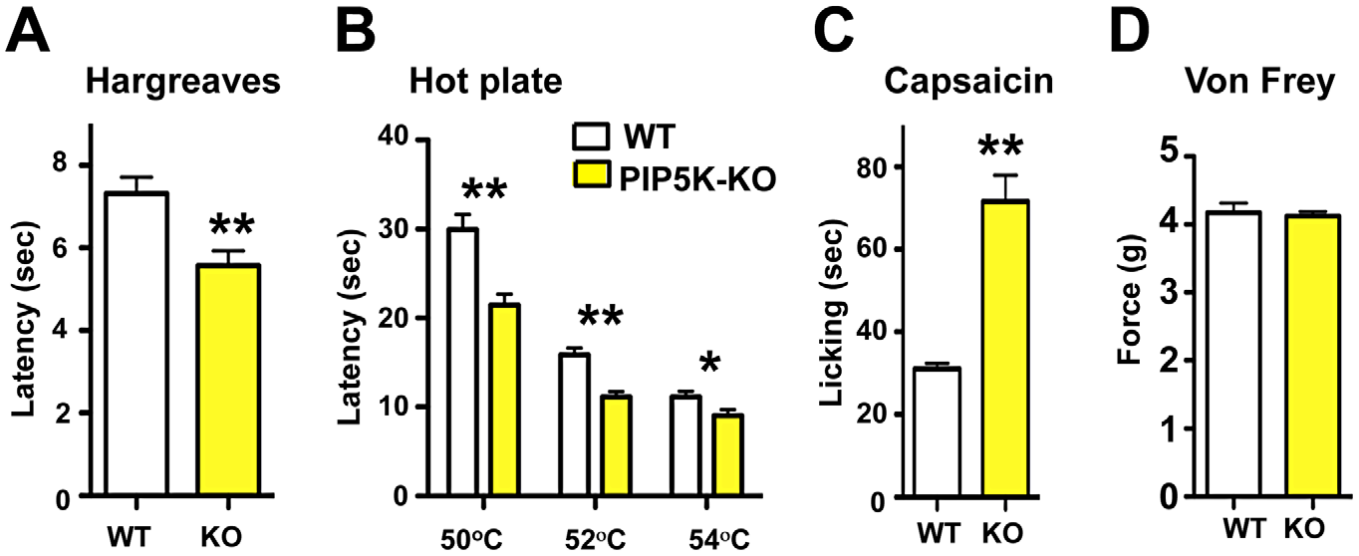
PI5Kα signaling controls thermal and capsaicin nociception *in vivo*. (A) Thermal pain thresholds of wild type (WT) and *PIP5Kα* ^−/−^ (KO) littermates in response to radiant heat (Hargreaves; n = 6 for WT; n = 6 for KO mice). (B) *PIP5Kα* KO mice also show enhanced thermal sensitivity in the hot plate assay (n = 12 for WT; n = 12 for KO mice) and (C) an exaggerated capsaicin-evoked behavioral response (n = 12 for WT; n = 9 for KO mice). (D) PIP5Kα KO mice exhibited normal mechanical pain (force threshold latency) as assessed by the von Frey test (n = 9 for WT; n = 9 for KO mice). All data are presented as mean +/− sem. *p<0.05; **p<0.01 (t-test).

Phosphatidylinositol signaling involves the generation of PIP2 via PIP5K, and then phosphorylation of PIP2 via PI3K to generate PIP3. PIP5Kα is highly expressed in the nervous system but no neuronal function for this kinase has been established [29]. *PIP5Kα* mutant mice are viable and exhibit an exaggerated anaphylactic immune reaction in response to Fc-receptor engagement [30]. We find that *PIP5Kα* mutant mice exhibit a significant hyper-responsiveness to radiant heat (Figure 2A) and contact heat, when compared to littermate controls (Figure 2B). In mammals, TRPV1 is the prototypical noxious heat thermo-receptor, and is also the receptor for capsaicin, the active ingredient in chili peppers [31]. We therefore tested whether *PIP5Kα* mutant mice exhibit exaggerated TRPV1 agonist responses. Indeed, following capsaicin injection, *PIP5Kα* mutant mice display heightened reactivity compared to littermate controls (Figure 2C) but exhibited no difference in mechanical pain threshold using the von Frey test (Figure 2D).

**Figure 3:**
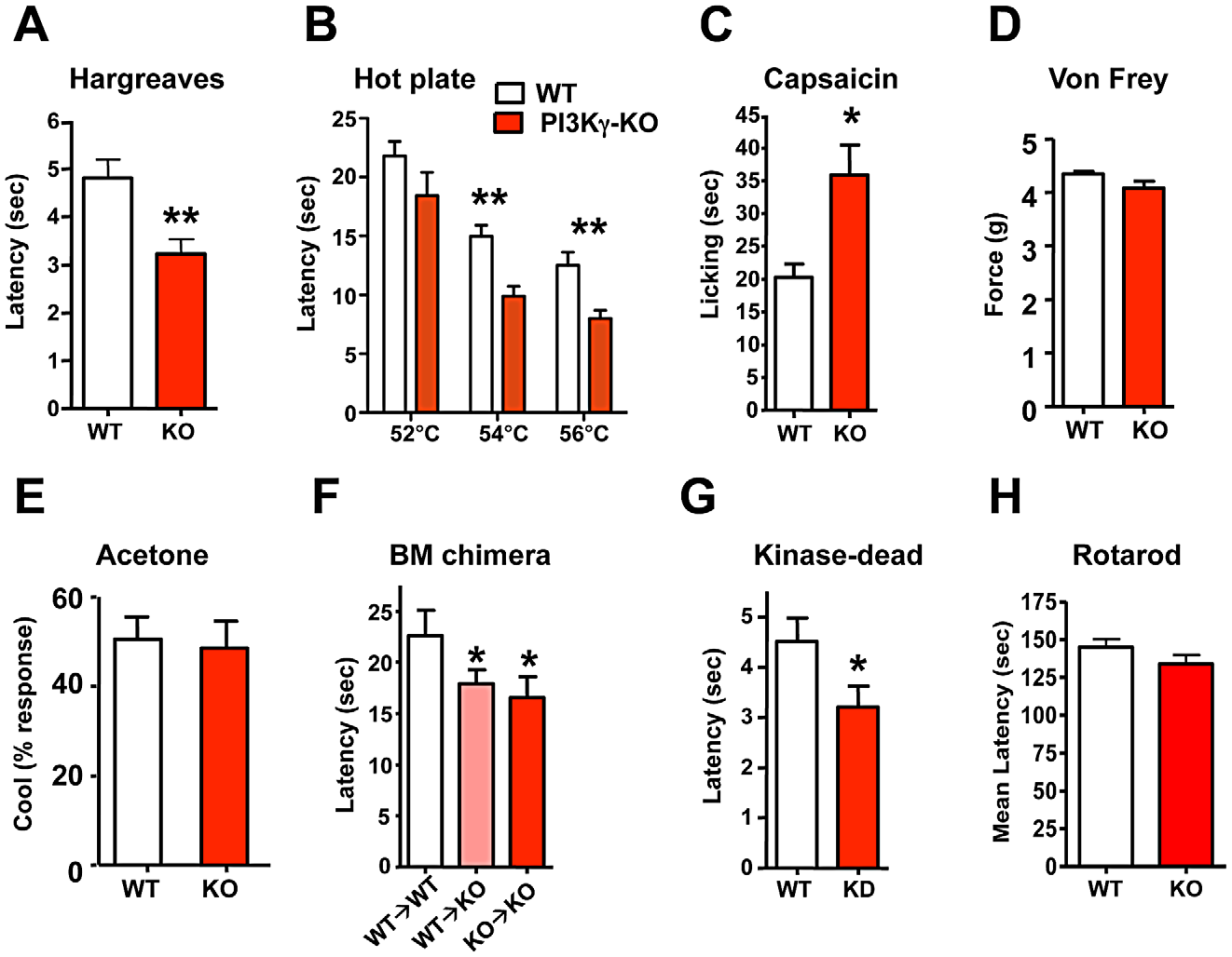
PI3Kγ signaling controls thermal and capsaicin nociception *in vivo*. (A) *PI3Kγ* ^−/−^ (KO) mice show enhanced thermal pain thresholds in response to radiant heat (Hargreaves test) as compared to wild type (WT) littermates (n = 22 for WT; n = 27 for KO mice). (B) *PI3Kγ* KO mice exhibit enhanced thermal sensitivity in the hot plate assay (n = 13 for WT; n = 9 for KO mice) and (C) an exaggerated capsaicin-evoked behavioral response (n = 9 for WT; n = 13 for KO mice). (D) Normal mechanical pain (force threshold latency) in *PI3Kγ* KO mice as assessed by the von Frey test (n = 21 for WT; n = 16 for KO mice). (E) *PI3Kγ* KO mice exhibit comparable cold sensitivity as assessed by acetone application (n = 21 for WT; n = 16 for KO mice). (F) Thermal nociception in *PI3Kγ* KO mice reconstituted with wild type (WT→KO) or *PI3Kγ* ^−/−^ (KO→KO) bone marrow. WT mice reconstituted with WT bone marrow are shown as controls. Mice were assayed using the hot plate assay at 54°C. n>6 for each group. (G) Thermal nociception thresholds in response to radiant heat (Hargreaves test) in littermate control and *PI3Kγ kinase-dead* (KD) knock-in mice (n = 19 for WT; n = 23 for KD mice). (H) Control and *PI3Kγ* KO mice exhibit similar coordination in the Rotarod test (n = 11 for WT and n = 12 for KO mice). All data are presented as mean +/− sem. *p<0.05; **p<0.01 (t-test).

PI3Kγ, the only G-protein coupled PI3K, is expressed in TRPV1-positive peripheral sensory neurons in both rats and mice [32], [33] and has been implicated in morphine-induced peripheral analgesia [32] and morphine tolerance [33]. Use of non-specific inhibitors, like wortmannin, has suggested a role for PI3' kinases in producing NGF-mediated TRPV1 sensitization [21],[34]. We therefore tested thermal nociception in PI3Kγ (p110γ) mutant mice [35] and found that these mice, like the *PIP5Kα* null mice, also exhibit an exaggerated behavioral response to radiant heat plantar stimulation using the Hargreaves test (Figure 3A). This enhanced pain sensitivity was confirmed using the hot plate assay (Figure 3B). *PI3Kγ* mutant mice also exhibit an enhanced pain response to a capsaicin challenge (Figure 3C). Similar to *PIP5Kα* null mice, the mechanical pain threshold using the von Frey test (Figure 3D), and the behavioral responses to acetone application (a cooling sensation) (Figure 3E) were comparable between control and *PI3K*γ^−/−^ littermates. Of note we found a similar thermal hyperalgesia phenotype in a second independent *PI3K*γ^−/−^ mutant mouse strain [36] (not shown). Thus, genetic loss of PI3Kγ and PIP5Kα results in enhanced pain responses to heat and capsaicin, providing evidence that phosphatidylinositol signaling acts as a negative regulator of heat pain perception and TRPV1 reactivity *in vivo*.

Since PIP5Kα and PI3Kγ cooperate to sequentially generate PIP3, and both mutant mice exhibit a similar hypersensitivity phenotype, we focused further on the role of PI3Kγ and PIP3 generation in setting the threshold for heat pain perception. PI3Kγ is highly expressed in haematopoietic cells and functions as key mediator of inflammatory cell migration to the site of injury [35], [37]. We therefore tested *PI3Kγ* mutant mice for potential defects in inflammation-induced pain sensitization, i.e. thermal hyperalgesia. *PI3Kγ^−/−^* and control mice developed comparable levels of thermal hyperalgesia (Figure S4A and S4B) following plantar CFA injection. CFA-induced inflammation, as determined by paw swelling, was also comparable between mutant and control mice (Figure S4C). To further exclude a potential role of haematopoietic cells, we transplanted wild type bone marrow into *PI3Kγ* mutant mice (WT→KO) and *PI3Kγ* mutant bone marrow into wild type mice (KO→WT). The presence of a wild type haematopoietic system did not rescue the enhanced sensitivity to thermal pain in the *PI3Kγ* mutant background (Figure 3F), i.e. the requirement for PI3Kγ in thermal sensing maps to non-haematopoeitic cells. PI3Kγ has been shown to act both in a kinase-dependent fashion, through conversion of PIP2 to PIP3, and in a kinase-independent manner [38]. We therefore tested the behavioral response of *PI3Kγ kinase-dead* (*KD*) knock-in mice. *PI3Kγ KD* mice exhibited a heightened reaction to noxious heat with a reduced thermal nociception latency (Figure 3G), comparable to the enhanced heat pain responses observed in complete *PI3Kγ* mutant mice. Thus, the kinase activity generating PIP3 modulates heat pain. We also assessed the general neurological phenotypes of *PI3Kγ^−/−^* mice, all of which appeared normal (Figure 3H; Figure S5A–S5G). Furthermore, the overall morphology and histology of the central nervous system appeared normal in *PI3Kγ^−/−^* mice. These data demonstrate that generation of PIP3 through PI3Kγ negatively regulates pain sensitivity *in vivo*.

**Figure 4:**
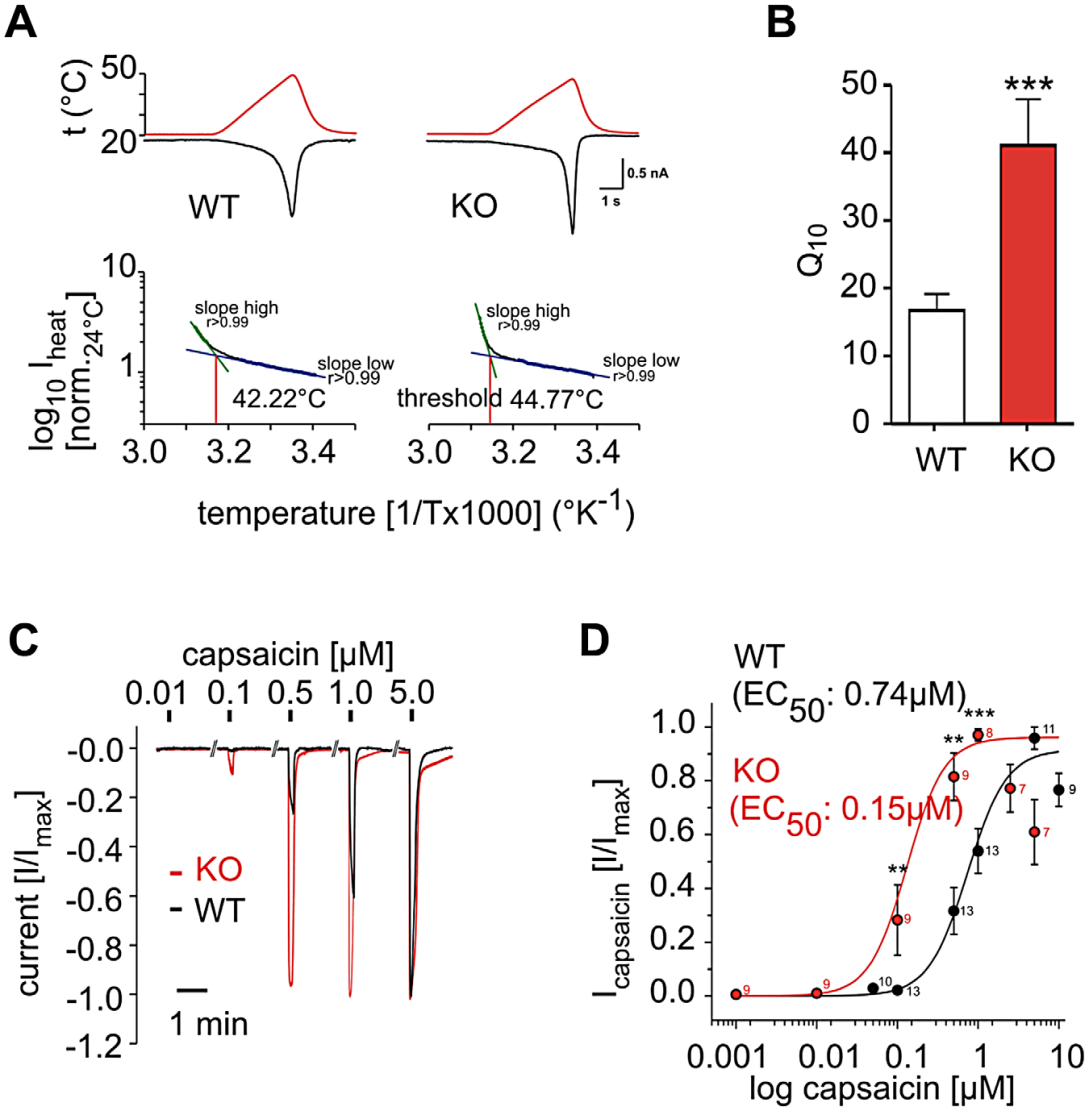
PI3Kγ acts in DRG neurons as a negative regulator of thermal and TRPV1 responses. (A) Representative temperature response ramps and Arrhenius plots for heat-activated currents measured in single DRG neurons isolated from wild type (WT) and *PI3Kγ* mutant (KO) mice. For temperature response ramps, red lines depict temperature ramps and black lines depict inward current. (B) Q10 as a measure of the rate of inward current changes in response to temperature. n = 37 for isolated WT; n = 9 for *PI3Kγ* KO DRG neurons. (C,D) Capsaicin sensitivity of DRG neurons isolated from WT and *PI3Kγ* KO mice. (C) Representative capsaicin responses from a single DRG neuron. (D) Dose-response curves to different concentrations of capsaicin. The capsaicin EC50 is indicated. Numbers indicate numbers of single neurons tested with the indicated capsaicin doses at the respective data points. Electrophysiology data was generated by single neuron patch clamping. Data are presented as mean +/− sem. ** p<0.01, *** p<0.001 (Mann-Whitney u-test).

To test if the phosphatidylinositol signaling pathway acts in primary sensory nociceptors, we employed electrophysiology on isolated wild type and *PI3Kγ^−/−^* dorsal root ganglion (DRG) neurons. *PI3Kγ^−/−^* DRG neurons responded to a thermal ramp (Figure 4A) with a significantly increased steepness in the inward current response to increasing temperature when compared to control neurons. This translated into a substantial increase in the Q10 value (Figure 4B), a measure of temperature-dependent rate change in channel conductivity, indicating that *PI3Kγ^−/−^* DRG cells exhibit massive hyper-activation in response to noxious heat, although initiation of this response occurs at a slightly elevated temperature (44.77°C for *PI3Kγ^−/−^* vs 42.22°C for wild type DRG neurons, Figure 4A). Since we also observed an enhanced response to capsaicin in *PI3Kγ^−/−^* mice *in vivo*, we directly tested TRPV1 reactivity to capsaicin in sensory neurons *in vitro*. In accordance with our behavioral data, isolated *PI3Kγ^−/−^* DRG neurons exhibited augmented sensitivity to capsaicin (Figure 4C and 4D). Thus, PI3Kγ functions as a negative regulator of TRPV1 responses in nociceptive neurons.

Our data provide a conserved functional systems network map for pain behaviour. This network map revealed many pathways and gene sets previously reported to be involved in mammalian nociception, including multiple genes annotated as candidate pain genes in the human OMIM database. Thus, our systems approach, starting from a functional whole genome fly screen and bioinformatic construction of a conserved pain network map, has the power to identify regulators of mammalian nociception. Our network pointed to a key role for phosphatidylinositol signaling in noxious heat nociception. Positive as well as negative regulatory functions of phosphatidylinositol signaling on the thermal nociceptive sensor TRPV1 have been reported [15], [16], [19], [20], [23], [24]. Our results provide genetic data that the phosphatidylinositol signaling pathway is relevant to heat pain sensitivity *in vivo*. In particular, we find that the lipid kinases PIP5α and PI3Kγ are involved in regulating heat nociception responses by acting as negative modulators of thermal pain perception and TRPV1 activity.

Our data reinforce the extraordinary evolutionary conservation of the neurobiological mechanisms of nociception, from its manifestation as an acute damage avoidance response in simple organisms like flies to the complex sensation of pain in mammals. When used in conjunction with additional complimentary approaches (e.g. published literature, gene expression profiling, or genetic association studies), this systems network map should be a valuable tool to further pinpoint and prioritize novel candidate nociception genes in mammals.

## Materials and Methods

### Bioinformatics analysis

Identification of fly orthologs in mouse and human was done using pre-computed orthology predictions [39]. Gene Ontology (GO) analysis was performed using GOstat. Binding partner identification was done using GeneSpring GX. Hypergeometric tests were used to identify over-represented gene lists (BROAD Institute) and pathways (KEGG) amongst the pain hits and to generate a conserved systems map. Pain genes and binding partners in the system map that have been annotated as pain genes in the Online Mendelian Inheritance in Man database or by our previous Microarray experiments [28] were also identified.

### Mouse behavioral tests

PI3Kγ (p110γ) knock out [35], [36], kinase dead PI3Kγ knock-in [38], and PIP5Kα mutant mice [30] and have been previously described. Thermal and mechanical sensitivities were assessed using the Hargreaves, hot plate, and von Frey tests. Capsaicin behavior was assessed over 5 minutes following intraplantar injection of capsaicin.

### Single DRG neuron recordings

Lumbar dorsal root ganglia (DRG) were harvested as previously reported [40], [41]. Patch-clamp recordings were performed using the whole-cell voltage-clamp configuration of the patch-clamp technique as previously described [40], [41].

### Ethics statement

All mice were bred and maintained according to an ethical animal license protocol complying with the current Austrian law.

Detailed Materials and Methods are available in Text S1.

## Supporting Information

Figure S1 Flow charts for global bioinformatics analyses. (A) Flow chart indicating the steps in the conversion of *Drosophila* candidate thermal nociception genes into mouse and human orthologs. (B) Flow chart indicating KEGG and C2 gene set analysis in *Drosophila*, mice, and humans. Data from all three organisms were pooled to generate a global network map. See Methods for details.
(PDF)

Figure S2 Conserved regulators of thermal nociception. GO enrichment analysis for human (A) and mouse (B) orthologs of *Drosophila* thermal nociception hits. GO terms were pooled into functional categories for data representation. Numbers indicate the counts of GO terms included in each functional category. These GO terms are grouped according to their parent terms - cellular components (CC), biological processes (BP), and molecular functions (MF). (C) Global C2 data set for mammalian orthologs. Functional classification of C2 gene sets (MsigDb, Broad institute) found enriched in mouse and human orthologs of our primary fly candidate thermal nociception genes and their first degree binding partners. The numbers indicate statistically significant C2 genes sets grouped into each functional category.
(PDF)

Figure S3 A global network map of thermal nociception based on primary hits without binding partners. The systems network includes data from the significantly enriched *Drosophila* KEGG pathways and GO processes, mouse and human KEGG pathways and C2 gene sets based on analysis of primary screening hits without binding partners. Pathways, processes and gene sets that share a role in a biological process were pooled into functional classes while the underlying genes that constitute them are depicted with a connection to their respective functional class. Functional classes (brown), genes representing hits with pain phenotype (red), and developmental lethal genes (blue) are shown in the network. For the entire list of individual pathways and gene sets see Tables S5, S6, S7.
(PDF)

Figure S4 PI3Kγ KO mice show no difference in inflammatory pain responses. (A) PI3Kγ KO mice exhibit significant enhancement of baseline thermal nociception sensitivity but a comparable degree of thermal hyperalgesia following intraplantar CFA challenge. (B) A sensitization ratio (Baseline/CFA latency) shows similar CFA-induced sensitization in wild type (WT) and PI3Kγ KO mice 10 days after CFA injection. (C) WT and PI3Kγ KO mice exhibit similar paw swelling in response to CFA over the course of the experiment (data shown was recorded at day 8). Data are presented as mean +/− sem. * P<0.05, ** P<0.01 by Student's t test.
(PDF)

Figure S5 Basic behavioral analysis of PI3Kγ KO mice. PI3Kγ expressing control and PI3Kγ knock-out (KO) mice exhibit similar behavioral responses in multiple paradigms. (A) PI3Kγ KO mice exhibit intact accuracy in a test for skilled reaching and (B) a slight decrease in cages crosses over a 24 hour period; but this trend failed to reach statistical significance. (C) In an open field test, control and PI3Kγ KO mice showed the same activity over a ten minute period and (D) similar levels of anxiety-related defecation. (E–G) No differences were detected between control and PI3Kγ KO mice in three models of learning: (E) Water maze, (F) T-maze, and (G) passive avoidance learning. (n = 11 for control and n = 12 for KO for the above tests). Data are presented as mean +/− sem. In all experiments there we no significant differences (t-test).
(PDF)

Table S1 Predicted mammalian orthologs for *Drosophila* pain genes and binding partners. To identify orthologs between *Drosophila* and mouse or *Drosophila* and human, we used pre-computed orthology predictions obtained from Compara r49, Homologene (03/08), Inparanoid v6.1, Orthomcl v2 [39]. Both One-to-one and many to many mappings were observed between the *Drosophila* and the mammalian genes.
(XLS)

Table S2 Drosophila candidate pain genes with known mammalian pain gene orthologs. Drosophila candidate pain genes were compared to known mouse pain genes, and overlap is shown. Unique *Drosophila* (CG number) and mouse (Entrez ID) identifiers are provided. In some cases, multiple independent transgenic RNAi fly lines targeting a particular *Drosophila* gene were found to exhibit a nociception defect, in which case all transformant ids are provided for a given Drosophila CG number. In some instances, a single candidate *Drosophila* pain gene was homologous to multiple known mammalian pain genes. Mammalian pain genes were identified using the Pain Genes Database [42].
(XLS)

Table S3 GO analysis for mammalian orthologs of *Drosophila* candidate pain genes. GO enrichment for all mouse and human orthologs corresponding to *Drosophila* candidate pain genes. Significant GO ids, corresponding GO terms, functional category, Biological Process (BP), Molecular Function (MF) and Cellular Component (CC), gene count, total gene count, and P value have been included. Since GO terms that lie at level 4 or below in the GO hierarchy tree convey more biological information, we excluded terms with more than 500 genes. Further, if both parent and several child terms are found significant, we manually curated the data and retained the term that contained the maximum overlapping genes with the pain hit list. These terms are highlighted in the mouse and the human lists separately. From these selected GO terms, those that denote related functions in biological systems were manually clubbed together and functionally annotated. This analysis is included in the column labeled “Manually assigned functional category”. A *P-value* of 0.1 was considered significant. “Count” and “Total” indicate the numbers of hits among all genes assigned to a defined GO term.
(XLS)

Table S4 Interactions between candidate pain genes and first degree binding partners. All interactions involving mammalian orthologs of *Drosophila* pain hit genes and their first degree binding partners are listed. Each row corresponds to a biological interaction between two proteins. Every interaction has two participating nodes: Source and Target. Gene symbols and CG IDs for all source and target nodes are listed. Classifications of all source and target nodes into pain hits (pain), binding partners (BP), and developmentally lethal phenotypes are provided. Potential mosue and human orthologs are presented for hits and binding partners. Both One-to-one and many to many mappings were observed between the *Drosophila* and the mammalian genes.
(XLS)

Table S5 Mammalian KEGG analysis. Enrichment of KEGG pathways for mouse and human orthologs corresponding to *Drosophila* candidate pain genes. The complete analysis for 202 mouse (top), and 209 human (bottom) KEGG pathways are shown. For each pathway, the KEGG database ID, pathway name, KEGG gene sets, CG/Entrez IDs for the mapped genes, numbers of genes assigned to each KEGG pathway, numbers of hits and their binding partners that map to the KEGG pathways, percentages of genes mapped among the total KEGG gene sets, and hypergeometric probabilities are listed. KEGG pathway ID numbers are presented and can be used to obtain complete list of genes included (see http://www.genome.jp/kegg/). Pathways that are over 90% enriched were manually annotated into functional groups and combined with C2 gene sets for visual representation in Figure 1 and Table S7.
(XLS)

Table S6 Mammalian C2 gene set analysis. Analysis for 687 mouse (top) and 680 human (bottom) C2 gene sets found significant at 99% probability (hypergeometric test). For each gene set, the C2 gene set name, gene symbols, counts for the total number of genes, pain hit orthologs, and percentages of genes mapped among the total C2 gene sets are given. C2 gene set names are presented and can be used to obtain complete list of genes included (see http://www.broadinstitute.org/gsea/msigdb/index.jsp). For constructing the systems map of nociception and uniform functional annotation as in the KEGG pathways, a unique list of significant C2 gene sets were combined from mouse and humans and manually grouped into similar functional categories, as the KEGG pathways. Combined information is provided in Table S7.
(XLS)

Table S7 Combined KEGG and C2 data for mammalian orthologs of *Drosophila* candidate pain genes and their binding partners. (A) The table represents a combined list of select significant GO terms, KEGG pathways and C2 gene sets from *Drosophila*, mouse, and human, used to construct the combined systems map shown in Figure 1. Selected significant KEGG pathways and C2 gene sets were manually grouped into uniform functional categories as shown in the column “Gene set categories”. Gene symbols, Entrez IDs and *Drosophila* CG IDs were extracted for each pathway or gene set, as appropriate. (B) Percentage of original pain hits and lethals that contribute to each of the significant enrichment of each gene set or pathway.
(XLS)

Table S8 Overlap of pain systems map with pain OMIM and microarray data. A comparison of genes in the global network map of thermal nociception with genes with a pain annotation in the OMIM database (http://www.ncbi.nlm.nih.gov/omim) and previous rat microarray expression profiling data generated from pain studies [28] showed the global network map of thermal nociception is enriched for genes implicated in mammalian pain. Included are the Drosophila gene CGID, mammalian gene symbol, the genes status as a direct hit (pain) or predicted binding partner (BP), and if this gene was identified as significantly regulated in the dorsal horn (DH) or dorsal root ganglia (DRG) following experimentally induced chronic pain. Genes with a pain annotation in the OMIM database are also identified.
(XLS)

Text S1 This file contains Detailed Materials and Methods and Supporting References.
(DOCX)
