# Supplementary figures and images for: Construction of a Global Pain Systems Network Highlights Phospholipid Signaling as a Regulator of Heat Nociception
Source: PLoS Genet. 2012 Dec 6;8(12):e1003071. doi: 10.1371/journal.pgen.1003071 (PMC3516557; doi:10.1371/journal.pgen.1003071)

**A**

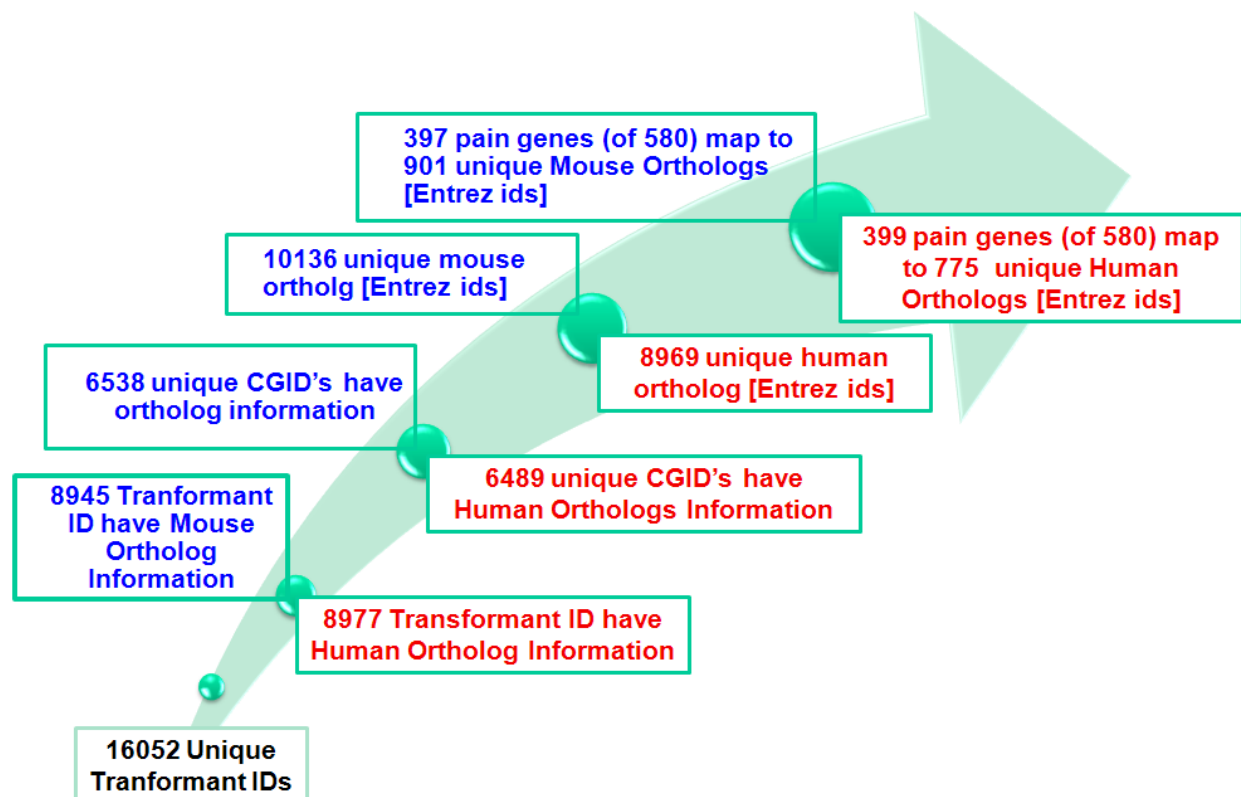

**B**

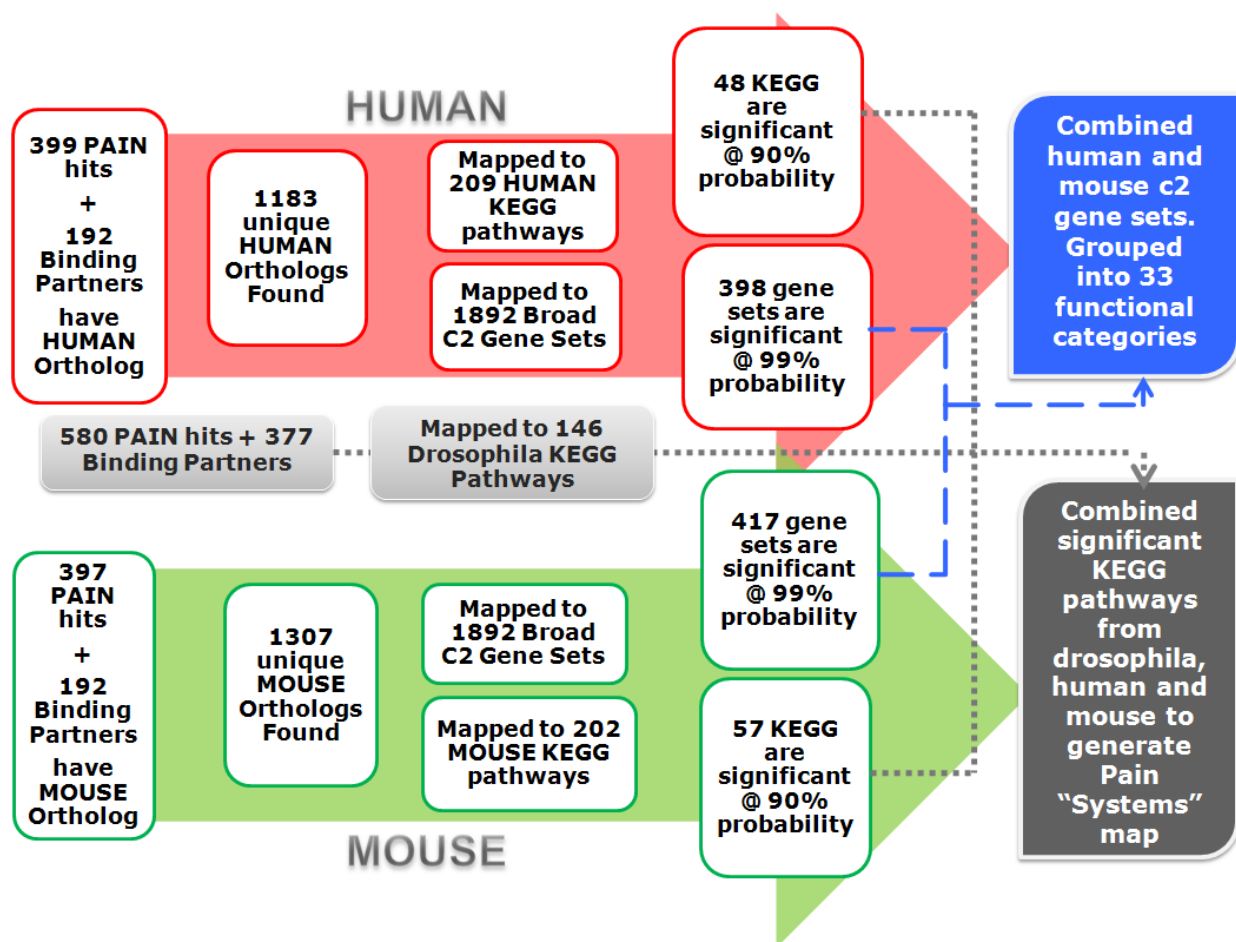

**Figure S1**

Supplement: Figure S1 — Flow charts for global bioinformatics analyses. (A) Flow chart indicating the steps in the conversion of Drosophila candidate thermal nociception genes into mouse and human orthologs. (B) Flow chart indicating KEGG and C2 gene set analysis in Drosophila, mice, and humans. Data from all three organisms were pooled to generate a global network map. See Methods for details. (PDF) [file pgen.1003071.s001.pdf]

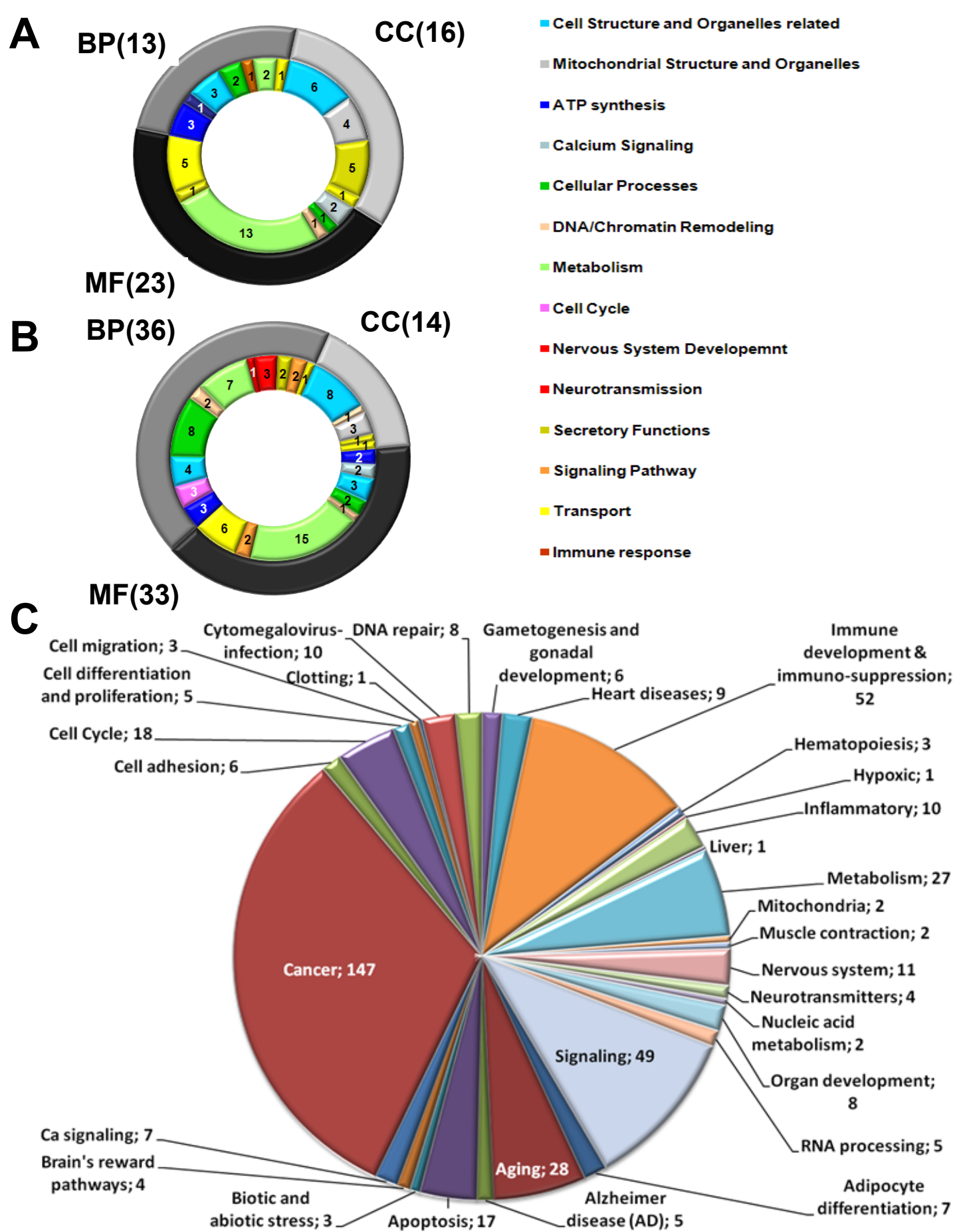

**Figure S2**

Supplement: Figure S2 — Conserved regulators of thermal nociception. GO enrichment analysis for human (A) and mouse (B) orthologs of Drosophila thermal nociception hits. GO terms were pooled into functional categories for data representation. Numbers indicate the counts of GO terms included in each functional category. These GO terms are grouped according to their parent terms - cellular components (CC), biological processes (BP), and molecular functions (MF). (C) Global C2 data set for mammalian orthologs. Functional classification of C2 gene sets (MsigDb, Broad institute) found enriched in mouse and human orthologs of our primary fly candidate thermal nociception genes and their first degree binding partners. The numbers indicate statistically significant C2 genes sets grouped into each functional category. (PDF) [file pgen.1003071.s002.pdf]

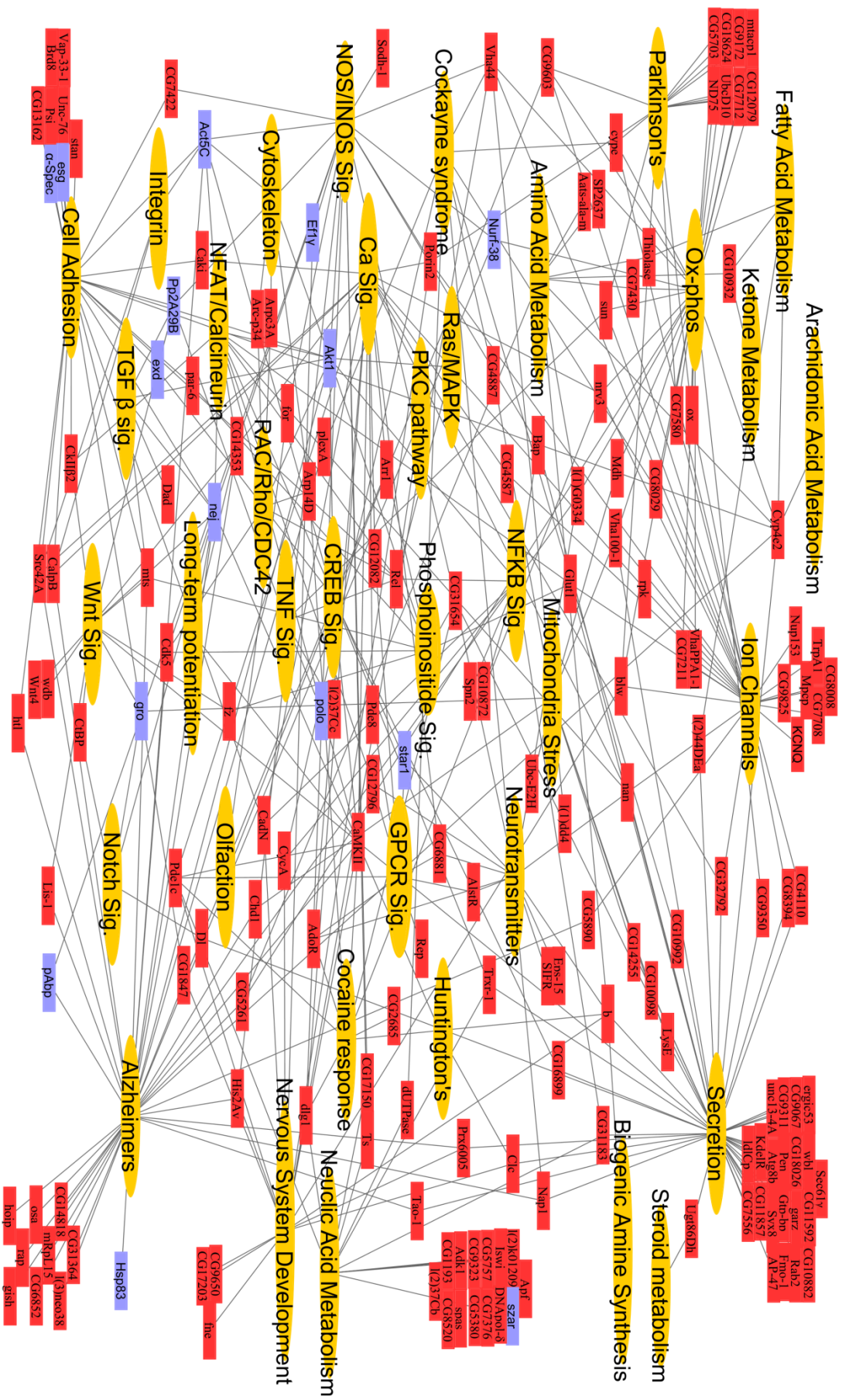

**Figure S3**

Supplement: Figure S3 — A global network map of thermal nociception based on primary hits without binding partners. The systems network includes data from the significantly enriched Drosophila KEGG pathways and GO processes, mouse and human KEGG pathways and C2 gene sets based on analysis of primary screening hits without binding partners. Pathways, processes and gene sets that share a role in a biological process were pooled into functional classes while the underlying genes that constitute them are depicted with a connection to their respective functional class. Functional classes (brown), genes representing hits with pain phenotype (red), and developmental lethal genes (blue) are shown in the network. For the entire list of individual pathways and gene sets see Tables S5, S6, S7. (PDF) [file pgen.1003071.s003.pdf]

**A**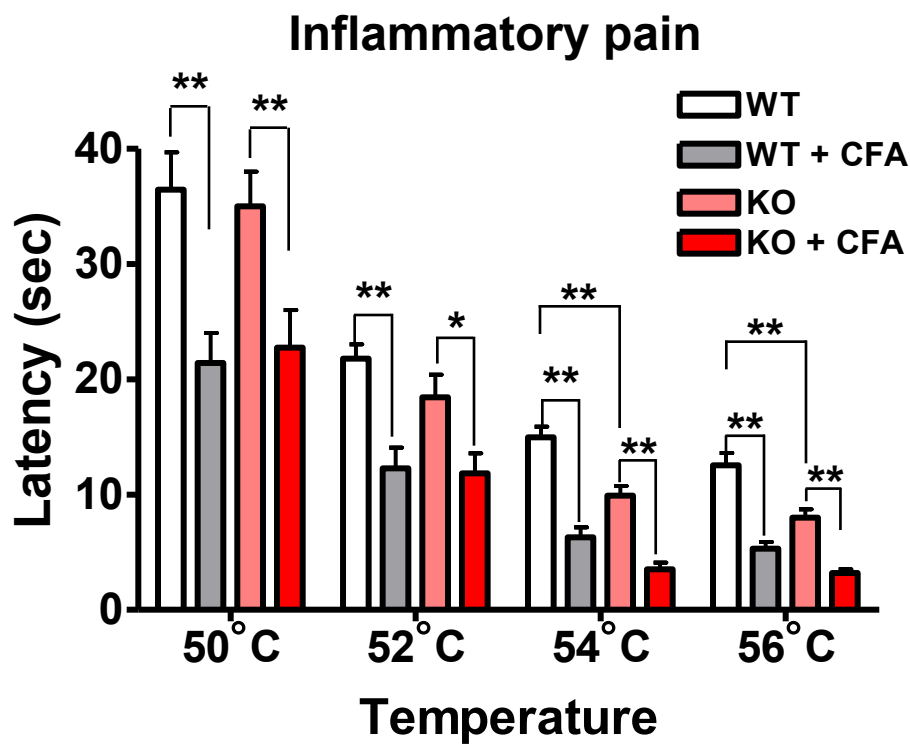**B**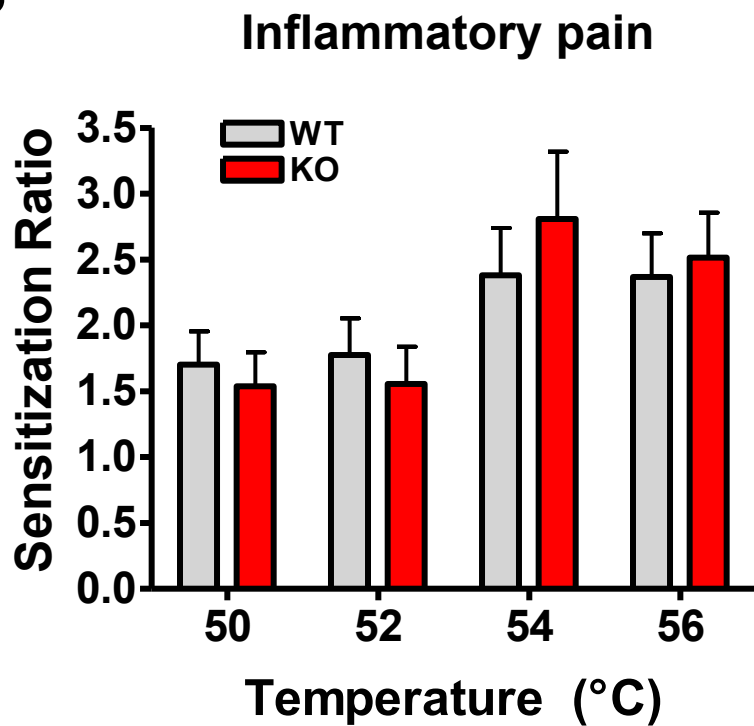**C**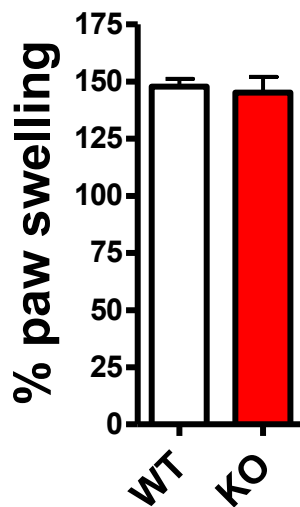**Figure S4**

Supplement: Figure S4 — PI3Kγ KO mice show no difference in inflammatory pain responses. (A) PI3Kγ KO mice exhibit significant enhancement of baseline thermal nociception sensitivity but a comparable degree of thermal hyperalgesia following intraplantar CFA challenge. (B) A sensitization ratio (Baseline/CFA latency) shows similar CFA-induced sensitization in wild type (WT) and PI3Kγ KO mice 10 days after CFA injection. (C) WT and PI3Kγ KO mice exhibit similar paw swelling in response to CFA over the course of the experiment (data shown was recorded at day 8). Data are presented as mean +/− sem. * P<0.05, ** P<0.01 by Student's t test. (PDF) [file pgen.1003071.s004.pdf]

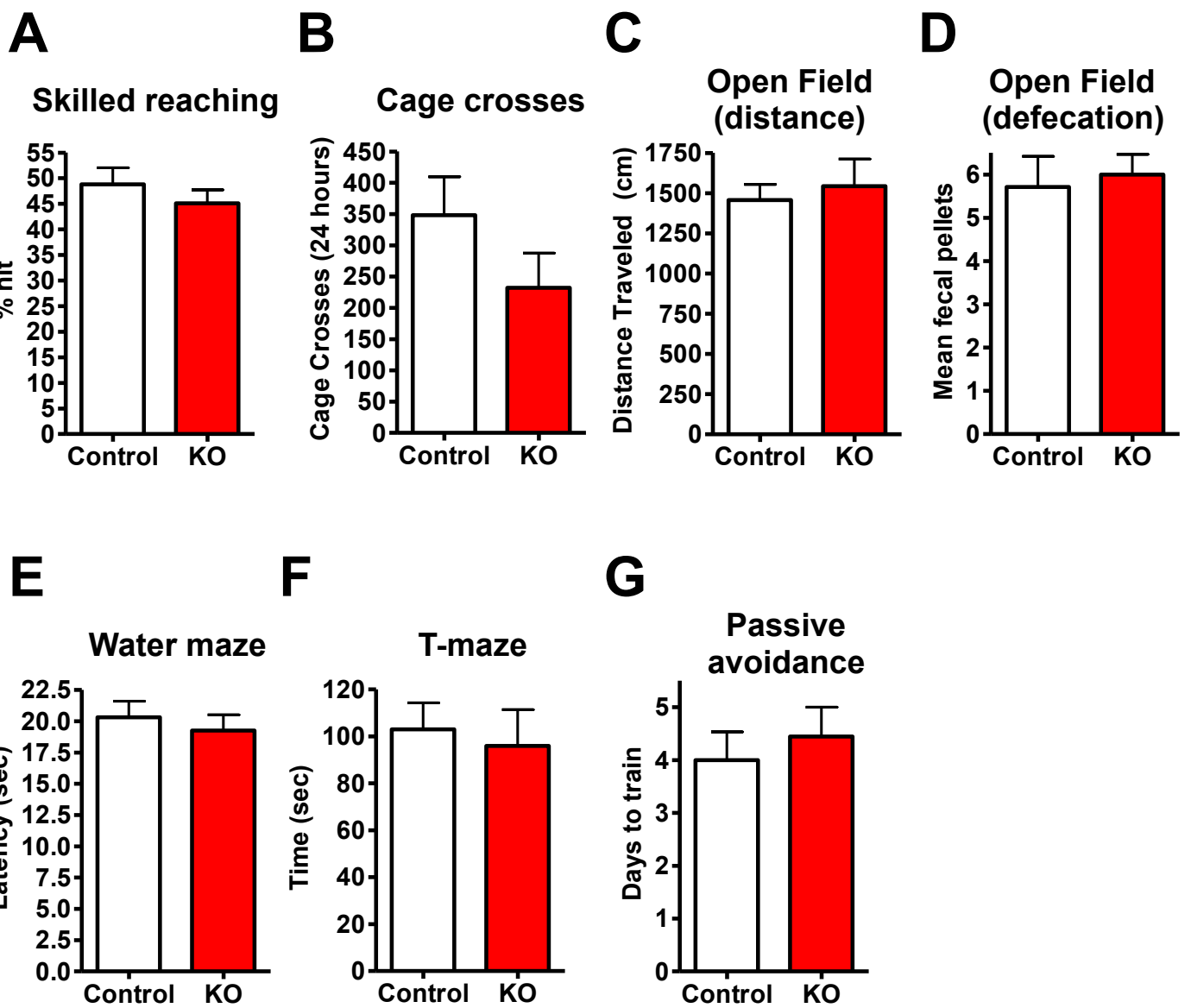

**Figure S5**

Supplement: Figure S5 — Basic behavioral analysis of PI3Kγ KO mice. PI3Kγ expressing control and PI3Kγ knock-out (KO) mice exhibit similar behavioral responses in multiple paradigms. (A) PI3Kγ KO mice exhibit intact accuracy in a test for skilled reaching and (B) a slight decrease in cages crosses over a 24 hour period; but this trend failed to reach statistical significance. (C) In an open field test, control and PI3Kγ KO mice showed the same activity over a ten minute period and (D) similar levels of anxiety-related defecation. (E–G) No differences were detected between control and PI3Kγ KO mice in three models of learning: (E) Water maze, (F) T-maze, and (G) passive avoidance learning. (n = 11 for control and n = 12 for KO for the above tests). Data are presented as mean +/− sem. In all experiments there we no significant differences (t-test). (PDF) [file pgen.1003071.s005.pdf]
