# Supplementary material for: Construction of a Global Pain Systems Network Highlights Phospholipid Signaling as a Regulator of Heat Nociception
Source: PLoS Genet. 2012 Dec 6;8(12):e1003071. doi: 10.1371/journal.pgen.1003071 (PMC3516557; doi:10.1371/journal.pgen.1003071)
Supplement: Text S1 — This file contains Detailed Materials and Methods and Supporting References. (DOCX) [file pgen.1003071.s014.docx]

**Text S1**

**Detailed Materials and Methods**

**Identification of fly orthologs in mouse and human.** To identify orthologs between *Drosophila* and mouse or *Drosophila* and human, we used pre-computed orthology predictions obtained from Compara r49, Homologene (03/08), Inparanoid v6.1, Orthomcl v2 [[1](#_ENREF_1)]. Since each database query outputs results using a different gene identifier, we transformed all the different identifiers to a single unique Entrez ID, for each gene. Both one-to-one, and many-to-many mappings were observed between the *Drosophila* and the mammalian genes. In cases where one *Drosophila* gene mapped to multiple mammalian orthologs, all the predicted orthologs of a given gene were considered for further downstream analysis. In cases where a mammalian gene of interest had multiple *Drosophila* orthologs, the fly gene with highest Z-score for the corresponding RNAi hit was mapped.

**GO classification.** Gene Ontology (GO) analysis was performed using GOstat (<http://gostat.wehi.edu.au/>). Default tool settings were used. GO analysis was run with mouse and human orthologs whose RNAi hits have a Z-score >1.65. Over- or under-represented (p<0.1) GO terms with corrections for multiple testing (Benjamini – Yekutieli correction) were identified, compared to the all mouse genes in Mouse genome index (mgi) and all human genes included in goa_human, respectively. Since terms that occur at a deeper level in the GO tree hierarchy, therefore containing lesser numbers of genes, are considered more biologically informative, we discarded terms containing more than 500 genes from further analysis. Significant GO terms were manually pooled and organized into “functional groups” based on their shared roles in a biological function, for visual representation of the GO data. A complete list of all GO terms and their assigned functional groups is provided in Table S3.

**Binding partner and pathway analyses.** Pathway analysis was performed using the *Drosophila* Pathway database in GeneSpring GX. Briefly, the Pathway database in GeneSpring GX contains binding partners from two sources: 1. those reported in open-source public databases like BIND and IntAct (IMEX consortium). The tool UI allows a query of the database to build networks of molecular relations (edges) amongst molecules (nodes) of interest. Data from the IMEX consortium only represent protein binding and promoter binding molecular interactions. Using filters in GeneSpring GX, we selected only binding molecular relations reported by the IMEX consortium to create this network, thereby including only experimentally proved physical interactions between the corresponding protein molecules.

**Hypergeometric enrichment test.** A hypergeometric test similar to the test used for GO enrichment analysis was used to identify over-represented gene lists (C2 from Msigdb, BROAD Institute) and pathways (KEGG) amongst the pain hits. The hypergeometric test considers only the percentage representation of genes corresponding to a biological pathway in the pre-computed pain gene list. This analysis was performed on the gene list identified as mouse or human orthologs corresponding to adult pain hits (Z-score > 1.65) in *Drosophila*.

**Generation of a systems map.** For the combined systems map, significant KEGG pathways, C2 gene sets and selected GO functional categories were manually grouped into uniform functional categories as shown in Table S7. Functional categories chosen for depiction were selected relevant to the biology of pain function. The complete list of functional categories is available in Tables S5 and S6. Of these, 37 functional categories were chosen for construction of the systems map. The list of these selected functional categories is available in Table S7. For each functional category, the corresponding genes that mapped to the KEGG pathways, C2 gene sets and GO term, included in the category were extracted. *Drosophila* orthologs for these genes were found and assigned to the category and visually represented into a systems map. For the construction of *Drosophila* KEGG pathway systems map, pathways rendered over 90% enriched by GSA analysis, were manually annotated into functional categories. Pain hit genes and their binding partners that belong to any pathway were extracted and assigned to the appropriate higher-level functional category. Data was represented as a systems map connecting these functional categories.

**Mouse behavioral tests.** PI3Kγ knock out [[2](#_ENREF_2)], kinase dead knock-in [[3](#_ENREF_3)], and PIP5Kα mutant mice [[4](#_ENREF_4)] are described. Baseline thermal and mechanical sensitivities were assessed using the Hargreaves and von Frey test (Ugo Basile Biological Research Apparatus Co., Comerio, Varese – Italy) following acclimation with the test apparatus. For paw withdrawal latencies, responses were determined by testing left and right paws. The hot plate test was done using a microprocessor-controlled unit (Ugo Basile). WT and PI3Kγ KO littermates or WT and PIP5Kα KO littermates were used as controls. Inflammatory pain was induced by intraplantar injection of Complete Freund's Adjuvant (CFA) (20 µl of a solution containing 5 mg of CFA in 10 ml of a 1:1 emulsion of saline and mineral oil) and behavior was tested on a hot plate as above. Paw swelling indicative of inflammation was evaluated using of a spring loaded caliper (Mitutoyo). Capsaicin behavior was assessed by time spent licking over 5 minutes following intraplantar injection of capsaicin (3 μg in 10 μl; dissolved in 5% ethanol, 5% Tween-80 and 90% saline; Sigma). For acetone-induced cold pain, a drop (50 μL) of acetone was placed against the centre of the plantar surface of the hindpaw and responses were recorded [[5](#_ENREF_5)]. Other behavioral test were as described previously [[6](#_ENREF_6)], Briefly, for skilled reaching task, each mouse was placed on a restricted diet and conditioned in the reaching box for 5 days. On the 6^th^ day mice were placed in the reaching box and reaching was scored for 5 minutes beginning with the first attempt. For circadian activity, mice were individually housed in cages with photosensors. The mice were monitored for 24 hours with a 12/12 light dark cycle and the number of cage crosses was recorded. For open field activity, mice were individually housed in clear plastic cages with infrared sensors and left for 10 minutes in conditions of low noise and dim light. Total horizontal activity was tracked and fecal pellets were counted at the end of the test. For rotorod, mice were trained for 3 days at very low speed and then tested while gradually increasing rotation speed. Mean latency on rod was recorded. For water maze, a 1.2 diameter milk pool was used with a 14 X 14 cm stationary platform hidden 7 mm beneath the liquid surface. Mice were placed in the pool in randomized quadrants and given 60 seconds to locate the safe platform. At the end of each trial mice that failed to locate the platform were placed on it for 10 seconds. After 14 trials over 7 days, the platform was moved to a new quadrant. Mean latency to find the platform is presented. For T-maze, mice were placed in a T shaped maze with one arm of the maze blocked off. On the second day the blocked arm was opened, and time spent in the new area was recorded. For passive avoidance, mice were placed in a clear plastic box with metal bars wired to distribute 0.12 milliamps built into the base. A black plastic platform was fixed in the center of the box. Mice were placed in the black “safe” platform, and when they step from the platform they were given a 1 second shock, and then removed from the apparatus. This process was repeated until mice no longer step from the safe platform.

**Bone marrow transplantations.** Six to eight week-old recipient mice underwent a lethal total-body irradiation (1000 Rad). Freshly isolated donor bone marrow cells were then injected into syngenic recipient mice (5 x 10^6^ cells per mouse) 24 hours after irradiation. PCR analyses of blood cells and tail DNA indicated that ~ 95% of blood circulating leukocytes were of donor origin.

**DRG neuron cultures***.* Lumbar dorsal root ganglia (DRG) were harvested, treated enzymatically with Liberase Blendzyme1 (Roche, Switzerland) and Trypsin-EDTA (Invitrogen, Austria), and dissociated mechanically with a fire-polished Pasteur pipette as previously reported [[7](#_ENREF_7),[8](#_ENREF_8)]. The isolated DRG cells were washed, plated on glass coverslips coated with poly-l-lysine/laminin (Sigma,) and cultured in synthetic serum-free medium (supplemented TNB™, Biochrom,) at 37 °C in 5% CO_2_.

**Patch-clamp recordings***.* Using the whole-cell voltage-clamp configuration of the patch-clamp technique, ionic currents were recorded from isolated DRG neurons at -80 mV holding potential as previously published [[7](#_ENREF_7),[8](#_ENREF_8)]. The external solution (ECS) contained (in mM) 145 NaCl, 5 KCl, 2 CaCl_2_, 1 MgCl_2_ (all Sigma), 10 glucose and 10 HEPES (Merck), at pH 7.3 adjusted with NaOH. Borosilicate glass micropipettes (Science Products, Germany) were filled with internal solution (ICS) containing (in mM) 138 Caesium methanesulfonate, 2 MgCl_2_, 2 Na_2_-ATP, 0.2 Na-GTP, 0.5 CaCl_2_, 5 EGTA (all Sigma) and 10 HEPES (Merck), at pH 7.3 adjusted with CsOH (Merck). After filling, electrode resistance was 3–5 MΩ. Currents were filtered at 2.9 kHz, sampled at 3 kHz and recorded using an EPC 9 and the Pulse v8.74 software (HEKA). Experiments were performed at room temperature and only one neuron was tested per Petri dish. An automated seven-barrel system with common outlet next to the cell under investigation (<100 µM) was used for fast drug administration and heat stimulation [[9](#_ENREF_9)]. Capsaicin was applied at different concentrations (0.001, 0.01, 0.1, 0.5, 1.0, 5.0 and 10 µM; Sigma; 1 mM stock solution solved in ethanol) for 10 seconds each. Heat-activated inward currents (I_heat_) were elicited by applying ramp-shaped heat stimuli at 120 s intervals with linear temperature increases from 25 to 50-55°C within 5 seconds. Current values were sampled at the rising phase of the temperature ramp, normalized to 24°C (bath temperature) and data represented as an Arrhenius plot [[10](#_ENREF_10)]. The temperature co-efficient Q_10_ was used to characterize temperature dependence of the membrane. In the linear range, the activation energy E_a_ was determined from the slope of the regression line (r>0.99) using the formula:

-E_a_ = 2.303Rlog_10_ (I_2_/I_1_)/((1/T_2_)-(1/T_1_))

R gas contant (8.314 JK^-1^mol^-1^)

I_1_ current at lower absolute temperature T_1_

I_2_ current at higher absolute temperature T_2_

The T_threshold_ was determined from the point of intersection of the two regression lines.

Q_10_ was calculated using the equation: Q_10_ = exp(10E_a_/(RT_1_T_2_)).

**Statistical analyses.** For mouse behavioral assays a Student’s t-test was used. For statistical analysis of electrophysiology data, a Mann-Whitney u-test was used. Unless otherwise indicated, data are represented as mean values ± sem.

**Supporting References**

1. Kuzniar A, van Ham RC, Pongor S, Leunissen JA (2008) The quest for orthologs: finding the corresponding gene across genomes. Trends Genet 24: 539-551.

2. Sasaki T, Irie-Sasaki J, Jones RG, Oliveira-dos-Santos AJ, Stanford WL, et al. (2000) Function of PI3Kgamma in thymocyte development, T cell activation, and neutrophil migration. Science 287: 1040-1046.

3. Patrucco E, Notte A, Barberis L, Selvetella G, Maffei A, et al. (2004) PI3Kgamma modulates the cardiac response to chronic pressure overload by distinct kinase-dependent and -independent effects. Cell 118: 375-387.

4. Sasaki J, Sasaki T, Yamazaki M, Matsuoka K, Taya C, et al. (2005) Regulation of anaphylactic responses by phosphatidylinositol phosphate kinase type I {alpha}. J Exp Med 201: 859-870.

5. Racz I, Nadal X, Alferink J, Banos JE, Rehnelt J, et al. (2008) Crucial role of CB(2) cannabinoid receptor in the regulation of central immune responses during neuropathic pain. J Neurosci 28: 12125-12135.

6. IQ Whishaw FH, B Kolb (1999) Analysis of Behavior in Laboratory Rodents. In: Johansson UWaH, editor. Modern techniques in neuroscience: Springer-Verlag, Berlin. pp. 1243–1275.

7. Obreja O, Rathee PK, Lips KS, Distler C, Kress M (2002) IL-1 beta potentiates heat-activated currents in rat sensory neurons: involvement of IL-1RI, tyrosine kinase, and protein kinase C. Faseb J 16: 1497-1503.

8. Obreja O, Biasio W, Andratsch M, Lips KS, Rathee PK, et al. (2005) Fast modulation of heat-activated ionic current by proinflammatory interleukin 6 in rat sensory neurons. Brain 128: 1634-1641.

9. Dittert I, Vlachova V, Knotkova H, Vitaskova Z, Vyklicky L, et al. (1998) A technique for fast application of heated solutions of different composition to cultured neurones. J Neurosci Methods 82: 195-201.

10. Vyklicky L, Vlachova V, Vitaskova Z, Dittert I, Kabat M, et al. (1999) Temperature coefficient of membrane currents induced by noxious heat in sensory neurones in the rat. J Physiol 517 ( Pt 1): 181-192.
